# Efficient Sorption of Arsenic on Nanostructured Fe-Cu Binary Oxides: Influence of Structure and Crystallinity

**DOI:** 10.3389/fchem.2021.840446

**Published:** 2022-01-20

**Authors:** Gaosheng Zhang, Zhijing Wu, Qianying Qiu, Yuqi Wang

**Affiliations:** Key Laboratory for Water Quality and Conservation of the Pearl River Delta, School of Environmental Science and Engineering, Ministry of Education, Guangzhou University, Guangzhou, China

**Keywords:** Fe-Cu binary oxide, arsenic, sorption, structure-performance relationship, crystallinity

## Abstract

To study the structure-performance relationship, a series of nanostructured Fe-Cu binary oxides (FCBOs) were prepared by varying synthesis conditions. The obtained binary oxides were well characterized using X-ray diffraction (XRD), transmission electron microscope (TEM), Brunner-Emmet-Teller (BET), magnetic and Zeta potential measurement techniques. Both As(V) and As(III) sorption on the FCBOs were evaluated by batch tests. Results show that the surface structure and crystallinity of FCBOs are greatly dependent on preparation conditions. The crystallinity of FCBOs gradually increases as the synthesis pH value increasing from 9.0 to 13.0, from amorphous phase to well-crystalline one. Simultaneously, the morphology change of FCBOs from irregular agglomerate to relatively uniform polyhedron has been observed. The sorption of arsenic is greatly influenced by the crystallinity and structure of FCBOs, decreasing with increasing degree of crystallinity. The amorphous FCBO has higher surface hydroxyl density than well-crystalline one, which might be the reason of higher sorption performance. As(V) is sorbed by the FCBOs via formation of inner-sphere surface complexes and As(III) is sorbed through formation of both inner- and outer-sphere surface complexes. This investigation provides new insights into structure-performance relationship of the FCBO system, which are beneficial to develop new and efficient sorbents.

## Introduction

Arsenic contamination has emerged as one of global environmental issues in the last decades due to its high toxicity (Smedley and Kinniburgh, 2002). In natural waters, arsenic exists mainly in two inorganic forms as arsenate [As(V)] and arsenite [As(III)]. In view of the serious adverse effects of arsenic, a number of treatment techniques such as coagulation/precipitation, ion exchange, adsorption, membrane filtration and biological treatment, have been exploited for arsenic removal from drinking water and wastewater. Among them, adsorption is commonly and extensively used, due to its simplicity in operation, high efficiency and cost-effectiveness (Mohan and Pittman, 2007; Hu et al., 2015). Various adsorbents have been developed and used for arsenic removal (Mohan and Pittman, 2007). Recently, metal (hydr)oxides such as Fe (hydr)oxides (Sowers et al., 2017; Usman et al., 2020), feroxyhyte (Usman et al., 2021), Al oxides (Han et al., 2013; Mertens et al., 2016), TiO_2_ (Guan et al., 2012; Yan et al., 2015), ZrO_2_ (Hristovski et al., 2008; Cui et al., 2012; Shehzad et al., 2019), CeO_2_ (Srivastava, 2010; Li et al., 2012), CuO (Martinson and Reddy, 2009; Yu et al., 2012; Reddy et al., 2013) etc., have attracted considerable attention because they exhibit strong sorption properties towards arsenic.

More recently, composite sorbents containing two or more metal oxides have been emphasized for their enhanced sorption performance and synergistic effect. For example, an Fe-Mn binary oxide prepared by Zhang and coworkers demonstrates a greater enhancement in both As(V) and As(III) removal (Zhang et al., 2014); amorphous Fe-Ti bimetal oxides synthesized by Rao and coworkers have higher performance in both As(V) and As(III) removal than pure component oxides (Rao et al., 2015); a Ce-Mn binary oxide synthesized by Chen and coworkers exhibits higher arsenic removal efficiency than parent oxides (Chen et al., 2018); an Fe-Ni-Mn trimetal oxide reported by Nasir and coworkers is found to be efficient for As(III) removal (Nasir et al., 2018); an Fe-Cu-Mn trimetal oxide fabricated by Zhang and coworkers displays high sorption capacity for both As(V) and As(III) (Zhang et al., 2020).

Many literatures demonstrated that the sorption ability of metal oxides was strongly affected by their structure and crystallinity. Therefore, it is very necessary to study systemically the structure-performance relationship of synthesized metal oxides. Some researchers have investigated this relationship of single metal oxide system (Jegadeesan et al., 2010; Dou et al., 2016). However, very few researches have been done about binary metal oxide systems (Dou et al., 2018). In our previous study, a novel amorphous Fe (III)-Cu (II) binary oxide prepared with a Cu/Fe molar ratio of 1:2 was found to have high sorption ability towards arsenic (Zhang G et al., 2013). Moreover, it was superior to its parent components (ferrihydrite and cupric oxide) and crystalline CuFe_2_O_4_ (Tu et al., 2012; Wei et al., 2019). This Fe(III)-Cu(II) binary oxide system is very interesting because it can exist as a pure compound of CuFe_2_O_4_ or a mixture of ferrihydrite and cupric (hydr)oxide, depending on preparation conditions (Tu et al., 2012; Zhang G et al., 2013), which is very different from other binary systems (Zhang et al., 2014; Rao et al., 2015). Therefore, to investigate the structure-performance relationship of Fe(III)-Cu(II) binary oxide system is very vital, which will facilitate further understanding the arsenic sorption behaviors on binary oxide system and developing new composite adsorbents. To our best knowledge, until now, no information about the structure-performance relationship of Fe (III)-Cu (II) binary oxide is available in literatures.

Therefore, the main objectives of this study are 1) to synthesize a series of Fe-Cu binary oxides with different structure and crystallinity degree by varying the synthesis solution pH; 2) to characterize the morphology and crystallinity of as-synthesized binary oxides using a variety of techniques; 3) to evaluate the arsenic adsorption behavior and performance of binary oxides by batch tests; and finally 4) to investigate the structure-performance relationship of Fe-Cu binary oxide system.

## Materials and Methods

### Materials

Analytical grade chemicals including FeCl_3_.6H_2_O, CuSO_4_.5H_2_O and NaOH were purchased from Sinopharm Chemical Reagent Beijing Co., Ltd. (Beijing, China). They were directly used and no further purification was done. As (V) and As (III) stock solutions were prepared with deionized water using Na_2_HAsO_4_.7H_2_O and NaAsO_2_, respectively. Arsenic working solutions were freshly prepared by diluting stock solutions with deionized water. Glass vessels were used as reactors. Before use, reactors were firstly cleaned using 1% HNO_3_ solution and then washed several times with deionized water.

### Preparation of Fe-Cu Binary Oxides (FCBOs)

A series of FCBOs with a Fe/Cu molar ratio of 2:1 were prepared at different pHs, according to a slightly modified method described by Zhang G et al. (2013). Specifically, about 10.8 g ferric chloride hexahydrate (FeCl_3_.6H_2_O) and 5.0 g copper (II) sulfate pentahydrate (CuSO_4_.5H_2_O) were dissolved in 400 ml deionized water. Under vigorous mechanical-stirring, NaOH solution (3 M) was added dropwise to raise the pH of mixture to a predetermined value (9.0 or 11.0 or 12.0 or 13.0). The formed suspensions were continuously stirred for 0.5 h, aged at 100°C for 6 h using a hot water bath. After cooling, the prepared suspensions were washed several times with distilled water. Afterwards, they were treated by filtration and dried at 55°C for about 24 h. The dried FCBOs were crushed into fine powders (0.5–50 µm) and stored in a desiccator. According to the synthesis pH value, these FCBOs are denoted as FC1, FC2, FC3, and FC4, respectively.

### Characterization of FCBOs

X-ray diffraction analyses were performed on a Rigaku D/Max-3A diffractometer using Ni-filtered copper Kα one radiation (XRD, Rigaku, Japan). The morphology of FCBOs was analyzed using a transmission electron microscope (TEM, Hitachi H-800, Japan). Specific saturation magnetization (*M*_s_) and magnetization remanence (*M*_r_) measure of particles’ magnetism, was determined using vibrating sample magnetometer at room temperature (VSM, Model 7,307 Lakeshore, United States). Specific surface area was determined by nitrogen adsorption (BET-method) using a surface area analyzer (Nova 2000e; Quantachrome Instruments, United States). A zeta potential analyzer (Zatasizer 2000; Malvern, UK) was used to analyze zeta potential of the FCBOs.

The density of surface hydroxyl sites was determined by a surface titration method (Dou et al., 2016). Fe-Cu binary oxide (0.3000 g) was added into 50 ml of 0.05 mol/L NaOH solution. Its accurate molar concentration was titrated using a 0.0500 mol/L Na_2_CO_3_ solution-calibrated HCl solution (0.0502 mol/L). After 4 h shaking at 130 rpm with the temperature maintained at 25°C, the mixture solution was passed through a 0.45 μm membrane. The filtrate was titrated using the HCl solution and residual NaOH in it was neutralized until pH up to 7.0, then the amount of surface hydroxyl can be calculated based on the amount of NaOH consumed.

### Batch Sorption Experiments

A series of batch experiments were performed to investigate the sorption of arsenic on FCBOs. A certain amount of FCBO was put into 100 ml glass vessels containing 50 ml arsenic solution of different concentrations. The vessels were then oscillated on a shaker at 170 rpm for 24 h. After reaction, all samples collected were filtrated using 0.45 µm membrane and then were analyzed for arsenic. More detailed description of sorption experiments can be seen in the Supplementary Material.

### Analytical Methods

Prior to arsenic analysis, the aqueous samples were diluted to a concentration below 100 μg/L, acidified with concentrated HNO_3_, and stored in acid-washed glass vessels. Arsenic concentration was determined using an inductively coupled plasma mass spectrometry machine (ICP-MS, ELAN DRC II, Perkin Elmer Co. United States).

## Results and Discussion

### Properties of the FCBOs

**FIGURE 1:**
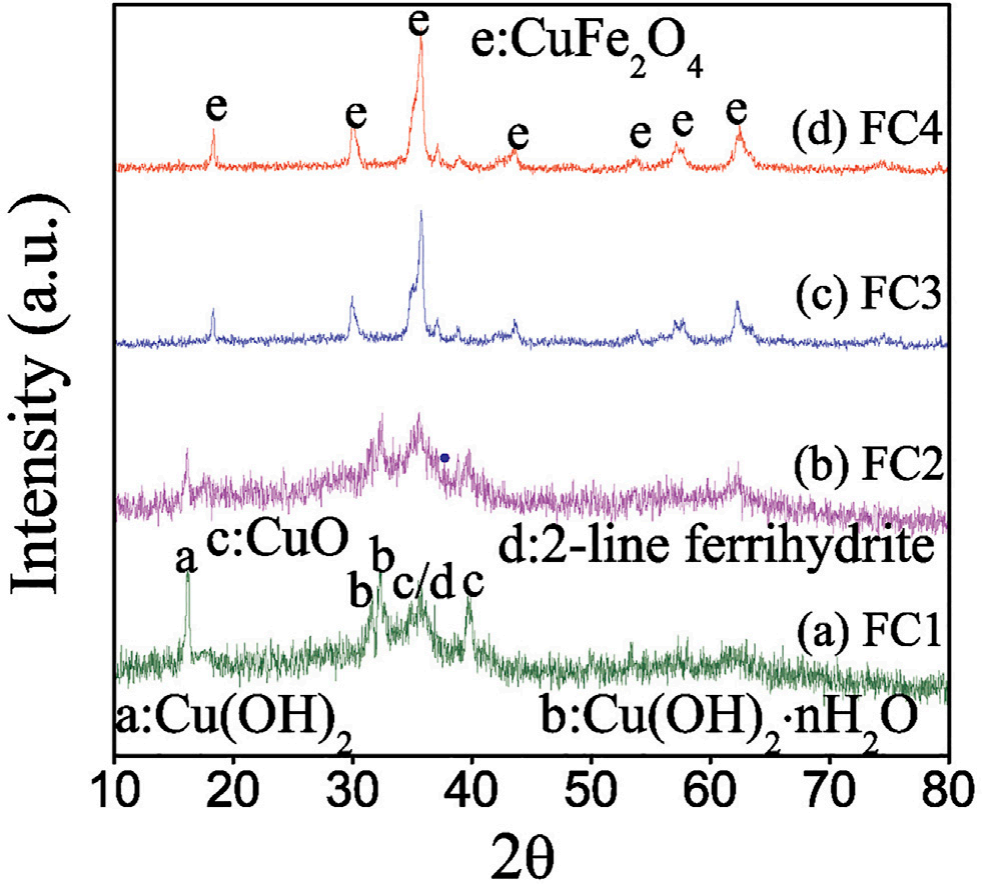
X-ray diffraction patterns of the Fe-Cu binary oxides: (a) FC1, (b) FC2, (c) FC3 and (d) FC4. (a: Cu(OH)_2_, b: Cu(OH)_2_·nH_2_O, c: CuO, d: 2-line ferrihydrite and e: CuFe_2_O_4_).

X-ray diffraction patterns of the as-prepared FCBOs are presented in Figure 1. For FC1, five broad peaks at approximately 16.2, 31.7, 32.3, 35.8 and 39.8° are observed. The characteristic peak at 16.2° belongs to the copper hydroxide (Cu(OH)_2_) (JCPDS 80-0656); the peaks at 31.7 and 32.3° are attributed to the hydrated copper hydroxide (Cu(OH)_2_·nH_2_O) (JCPDS: 42-0638); the peak at 35.8° might be ascribed to both copper oxide (CuO) (JCPDS: 45-0937) and 2-line ferrihydrite formation (Hofmann et al., 2004); the peak at 39.8° might belong to the CuO (JCPDS: 45-0937). Obviously, the FC1 exists mainly as an amorphous mixture of 2-line ferrihydrite and copper (hydr)oxides. The X-ray diffraction pattern of FC2 is closely similar to that of FC1. However, the intensity of peaks belonging to copper hydrated hydroxide, hydroxide and oxide decreases with an increase in synthesis pH value. When the synthesis pH reaches 12.0 (FC3), these peaks disappear almost completely, and new diffraction peaks appear at 18.3, 30.0, 35.8, 43.6, 53.7, 57.8 and 62.4°, respectively. These characteristic peaks are attributed to the well-crystalline copper ferrite (CuFe_2_O_4_) (JCPDS: 34-0425). With a further increase in synthesis pH value from 12.0 to 13.0 (FC4), the intensity of these peaks increases, indicating a greater crystallinity.

**FIGURE 2:**
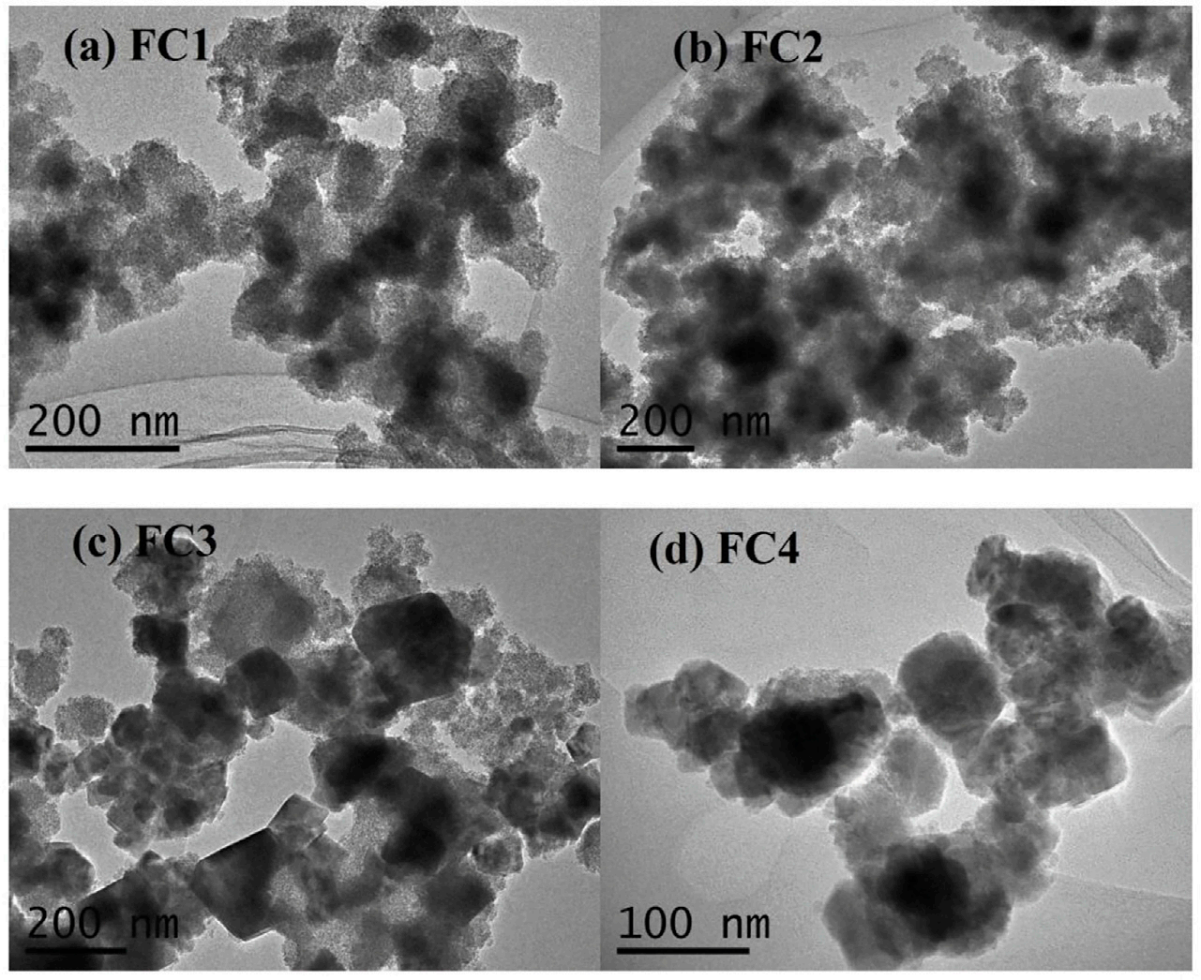
TEM images of the Fe-Cu binary oxides: **(A)** FC1, **(B)** FC2, **(C)** FC3 and **(D)** FC4.

Transmission electron micrographs (TEMs) of the FCBOs are shown in Figure 2. It can be seen that the FCBO particles produced at pH 9.0 (FC1, Figure 2A) are agglomerates of smaller nanoparticles. The morphology of FC2 (Figure 2B) is very similar to that of FC1. When the synthesis pH increases from 11.0 to 12.0, the major of amorphous particles (FC3, Figure 2C) becomes crystallized grains with visible grain boundaries and polyhedron shapes. For FC4 (Figure 2D), much more uniform grains are observed, suggesting well-crystalline CuFe_2_O_4_ particles are dominant under this condition. These results agree with those of XRD analysis.

**FIGURE 3:**
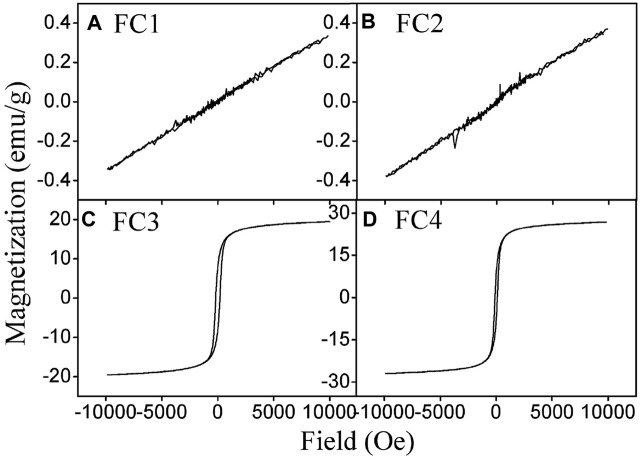
Magnetization loops of the prepared Fe-Cu binary oxides: **(A)** FC1, **(B)** FC2, **(C)** FC3 and **(D)** FC4.

The magnetic hysteresis curves of FCBOs are depicted in Figure 3. The hysteresis loops of FC3 and FC4 show a normal *S*-shape type, while those of FC1 and FC2 exhibit a nonhysteresis straight line, indicating that they are paramagnetic or superparamagnetic. The parameters of magnetic properties are summarized in Table 1. It can be seen that the saturation magnetization (*M*_s_) increases with increasing synthesis pH value. For FC1 and FC2, they demonstrate very weak magnetism and the value of saturation magnetization is less than 0.4 emu/g. As the synthesis pH increases from 11.0 to 12.0, the saturation magnetization of FC3 rises sharply to 19.5 emu/g, which is far higher than that of FC2 and is very close to the CuFe_2_O_4_ nanoparticle (20.6 emu/g) synthesized via citrate-nitrate combustion method (Anandan et al., 2017). The magnetism of FC4 is the strongest and the saturation magnetization is as high as 26.9 emu/g. These results indicate that the magnetic properties of the FCBOs are closely related to their crystallinity.

**TABLE 1:** Saturation magnetization and BET specific surface area of the as-prepared Fe-Cu binary oxide samples.

Samples	Synthesis pH	Saturation magnetization (emu g^−1^)	Specific surface area (m^2^ g^−1^)
FC1	9.0	0.34	268
FC2	11.0	0.37	202
FC3	12.0	19.52	130
FC4	13.0	26.88	90

The results of BET surface area measurements of the FCBOs are listed in Table 1. It can be clearly seen that the BET surface area of FCBOs decreases gradually with an increase in synthesis pH value. The BET surface area of FC1 is 268 m^2^/g. However, the surface area of FC4 is only 90 m^2^/g. It seems that the BET surface area of FCBOs is inversely proportional to its crystallinity.

### Sorption Envelope

**FIGURE 4:**
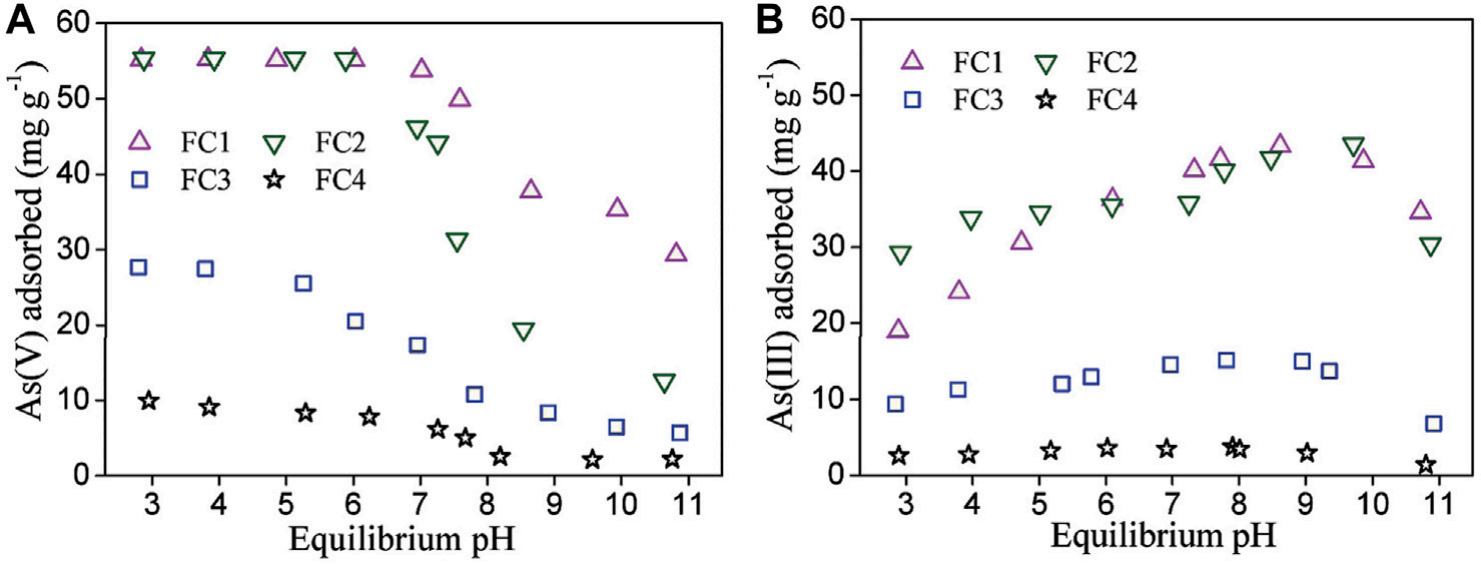
Effect of solution pH on **(A)** As (V) and **(B)** As (III) sorption by Fe-Cu binary oxides. Initial arsenic concentration = 11.0 mg/L, adsorbent dose = 200 mg/L and T = 25 ± 1°C.

The influence of solution pH on arsenic sorption was investigated and the results are shown in Figure 4. For all FCBOs, the sorption of As (V) depends evidently on solution pH value. The greatest sorption occurs under acidic conditions and decreases with increasing solution pH, which is a typical characteristic for anions sorption by metal oxides and oxyhydroxides (Ren et al., 2011; Jordan et al., 2014). Over the tested pH range (3.0–11.0), As(V) mainly exists as negatively charged H_2_AsO_4_^−^ and HAsO_4_^2-^ in water (pKa of dissociation is 2.20, 6.97 and 11.53, respectively). Under weak acidic conditions, the surface of the FCBO is positively charged because of protonation and H_2_AsO_4_^−^ is dominant species in aqueous solution, which is beneficial for electrostatic attraction between the surface of FCBO and the aqueous H_2_AsO_4_^−^. With an increase in solution pH, the surface of FCBO becomes less positively charged and even negatively charged. At the same time, HAsO_4_^2-^ (a more negatively charged As (V) species) becomes to be dominant. Therefore, the attraction between the surface of FCBO and As (V) species weakens and as a consequence, As (V) sorption decreases.

Compared to As (V), the influence of solution pH on As (III) sorption is markedly different. Its sorption enhances gradually as solution pH increases and a maximum sorption occurs at about pH 9.1. Afterwards, further increase in pH decreases the sorption of As (III). Similar phenomena have been reported for the As (III) sorption by other binary metal oxides (Ren et al., 2011). Generally, a maximal sorption of weak acid anions onto metal oxides occurs at pH values close to p*k*_a1_ of the acid. The p*k*_a1_ of H_3_AsO_3_ is 9.2. The reduction in As (III) sorption at pH above 9.1 may be ascribed to the Coulombic repulsion between the negative surface of FCBOs (pH_pzc_ = 7.3-9.0) and negatively charged As (III), whereas the predominant form of As (III) species is H_2_AsO_3_^−^.

### Sorption Isotherm

**FIGURE 5:**
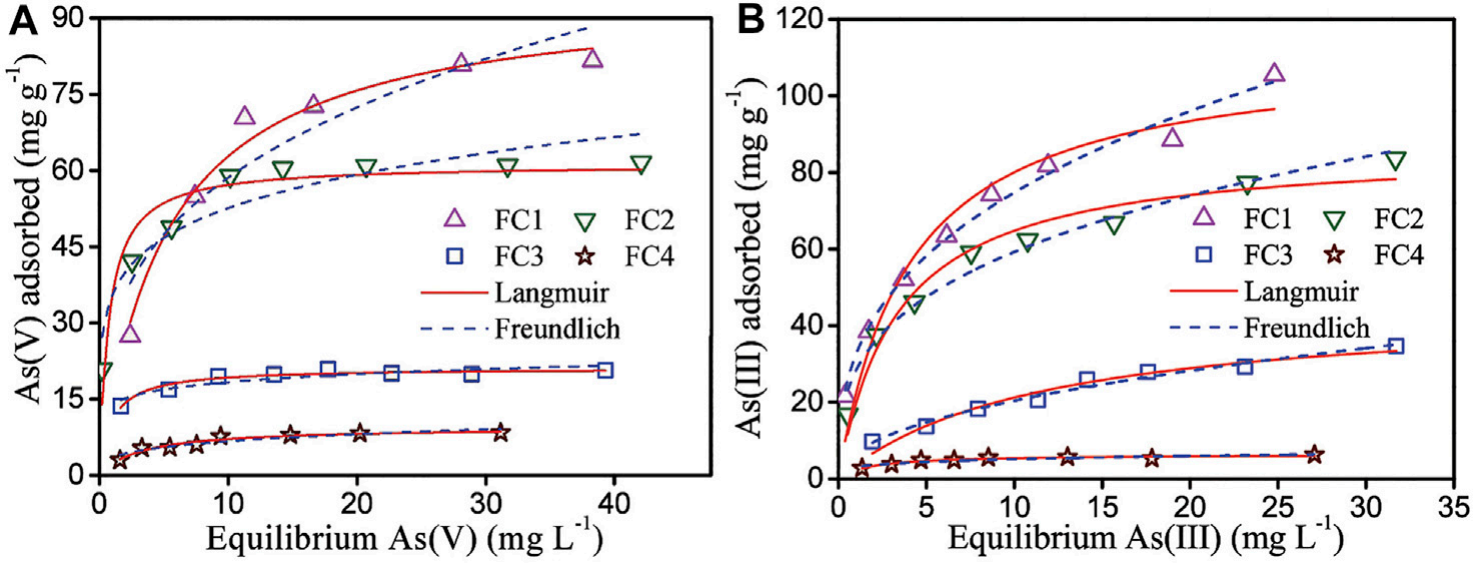
Adsorption isotherm of **(A)** As (V) and **(B)** As (III) by Fe-Cu binary oxides. Initial arsenic concentration = 2–60 mg/L, adsorbent dose = 200 mg/L, pH = 7.0 ± 0.1 and T = 25 ± 1°C.

The sorption isotherms of arsenic on the FCBOs are depicted in Figure 5. Obviously, the sorption of arsenic by the FCBO is closely related with its crystallinity. The FC1 has the strongest uptake ability for both As(V) and As(III) and the maximum sorption capacities are 83.3 and 112.2 mg/g at pH 7.0 (Langmuir model), respectively. The arsenic uptake ability of FCBOs decreases in the following order: FC1>FC2>FC3>FC4. Furtherly, the maximum sorption capacities of FC1 and FC2 are far larger than those of FC3 and FC4. The sorption performance of metal oxides depends generally on their surface area. However, the decrease in arsenic uptake by the FCBOs is not completely proportional to the reduction of specific surface area. This can be explained as follows. The specific surface area of the FCBOs is determined by N_2_ molecule, which is smaller than the arsenic molecule. Partial surfaces of the FCBOs are inaccessible to arsenic molecule. Additionally, the As(V) and As(III) maximal sorption capacities of Fe-Cu binary oxide synthesized at pH 7.5 are 82.7 and 122.3 mg/g, respectively (Zhang G et al., 2013). Obviously, the arsenic sorption ability of FC1 is to very close to that of Fe-Cu binary oxide prepared under neutral condition.

Both Langmuir and Freundlich models (seen in the Supplementary Material) were employed to fit the isotherm data. The fitting results and obtained parameters are shown in Figure 5 and Table 2, respectively.

**TABLE 2:** Langmuir and Freundlich isotherm parameters for As (V) and As (III) adsorption on Fe-Cu binary oxides at pH 7.0 ± 0.1.

Adsorbent	As species	Langmuir model			Freundlich model		
		*q*_max_ (mg/g)	*K*_L_ (L/mg)	*R* ^2^	*K*_F_ (mg^1-1/n^L^1/n^g^−1^)	1/n	*R* ^2^
FC1	As(V)	83.3	0.19	0.977	58.9	0.31	0.844
FC2	As(V)	61.3	1.40	0.900	35.5	0.17	0.884
FC3	As(V)	21.1	1.03	0.935	14.0	0.12	0.806
FC4	As(V)	9.5	0.31	0.935	3.5	0.28	0.842
FC1	As(III)	112.2	0.25	0.936	32.6	0.36	0.988
FC2	As(III)	86.5	0.30	0.961	28.4	0.32	0.974
FC3	As(III)	45.3	0.09	0.959	6.9	0.47	0.980
FC4	As(III)	6.4	0.55	0.920	3.1	0.22	0.819

For As (V), the Langmuir model is more favorable for fitting the data, giving higher correlation coefficients (*R* ^2^). However, the Freundlich model is more suitable to describe the adsorption of As(III) on Fe-Cu binary oxides except for FC4, according to the correlation coefficients. The As (V) adsorption is likely a monolayer adsorption because the Langmuir model supposes that the adsorption process is a monolayer adsorption. While As (III) adsorption is a multilayer adsorption since the Freundlich model presumes that adsorption occurs on the heterogeneous surface and follows multilayer adsorption.

### Relationship Between Surface Hydroxyl Concentration and Arsenic Adsorption Capacity

**FIGURE 6:**
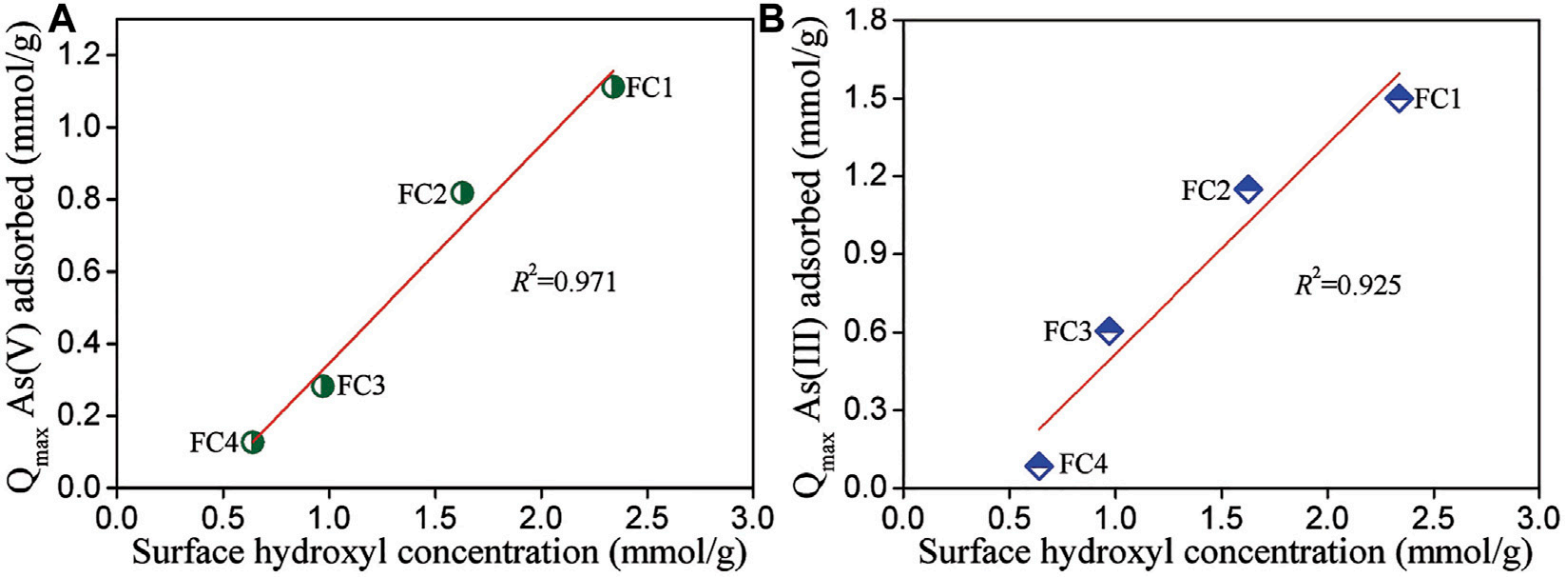
Relationship between surface hydroxyl concentration and **(A)** As (V) and **(B)** As (III) adsorption capacities of Fe-Cu binary oxides.

The surface of metal oxides in water is easily hydroxylated, due to the dissociation of chemisorbed water molecules, and the formed surface hydroxyl groups are responsible for anions adsorption from water by the exchange with hydroxide ions (Tamura et al., 1999). To reveal the relationship between surface hydroxyl concentration and arsenic adsorption capacity of FCBOs, the amounts of surface hydroxyl groups were determined by titration method and the results are demonstrated in Figure 6. It can be seen that FC1 has the largest amount of surface hydroxyl groups per unit weight (2.34 mmol/g), followed by FC2 (1.63 mmol/g), FC3 (0.97 mmol/g), and FC4 (0.64 mmol/g). Evidently, surface hydroxyl concentration of FCBO is negatively correlated with its crystallinity. However, arsenic sorption capacity of FCBO is well positively correlated with its surface hydroxyl concentration. The data was fitted using a linear equation and the coefficient of determination (*R* ^2^) of linear regression for As (V) and As(III) is 0.971 and 0.925, respectively. It should be noted that the intercept is negative, indicating that not all surface hydroxyl groups are efficient for arsenic, especially for FCBO with high crystallinity. This could be explained as follows. The space structure of arsenic species (H_2_AsO_4_^−^, HAsO_4_^2-^ or H_3_AsO_3_) is remarkably larger than that of hydrogen ions, which was used to determine the amounts of surface hydroxyl groups. Therefore, partial hydroxyl groups on the surfaces could not be available for the arsenic molecules. To some extent, the arsenic sorption capacity of FCBO could be evaluated by the amounts of surface hydroxyl groups.

### Zeta Potential and FTIR Analysis Before and After Arsenic Adsorption

**FIGURE 7:**
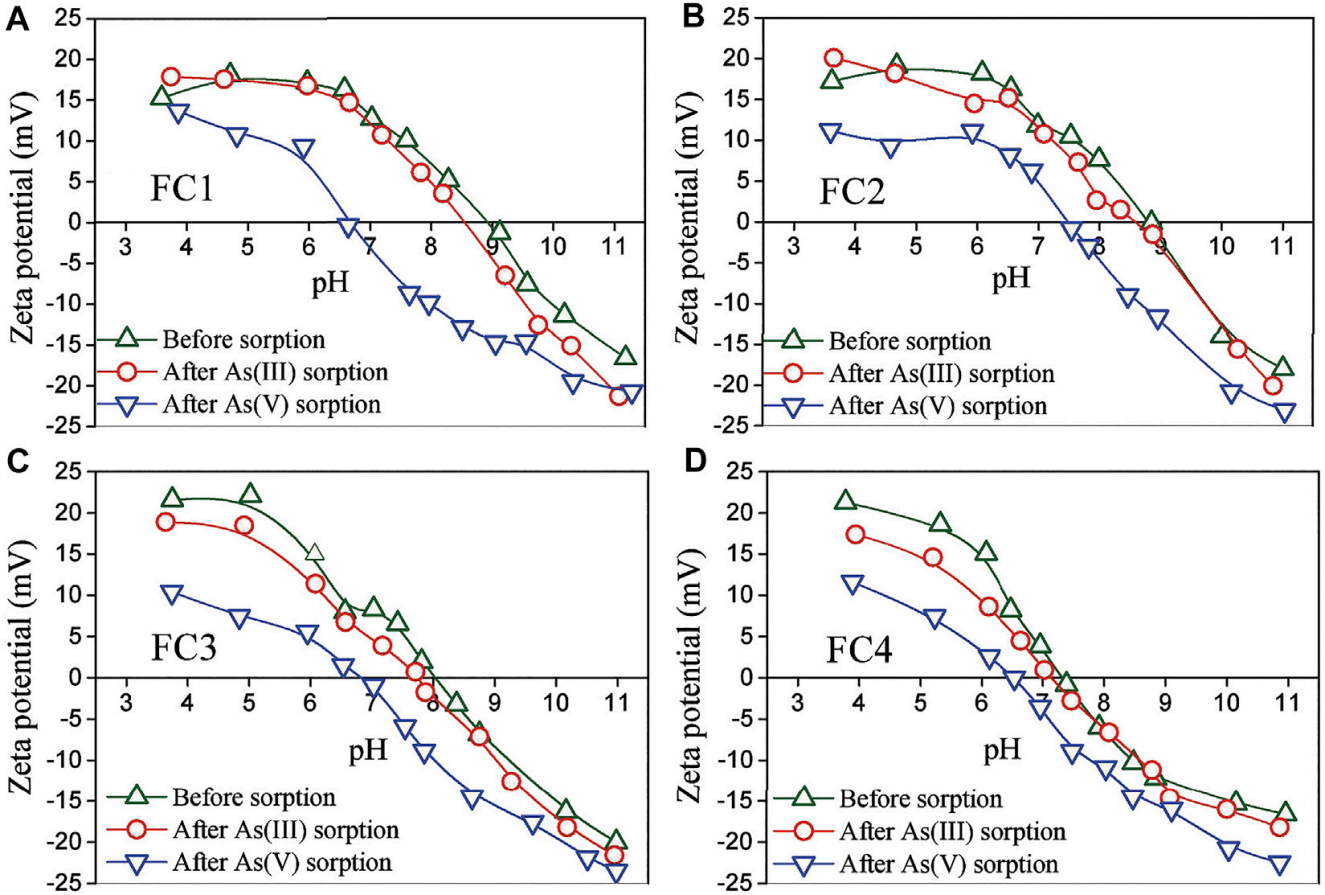
Zeta potential of Fe-Cu binary oxides before and after arsenic adsorption. Initial arsenic concentration = 10 mg/L, adsorbent dose = 200 mg/L, ionic strength = 0.01 M NaNO_3,_ equilibrium time = 72 h.

The zeta potentials of synthesized Fe-Cu binary oxides before and after reaction with arsenic were measured. As presented in Figure 7, the pH_pzc_ (pH at point of zero charge) of the virgin FC1, FC2, FC3 and FC4 were about 9.0, 8.8, 8.1 and 7.3, respectively. Evidently, the pH_pzc_ of the FCBOs decreases with increasing in crystallinity. This could be explained as follows. The pH_pzc_ values of CuO and Cu(OH)_2_ are commonly over 9.2 (Martinson and Reddy, 2009; Kosmulski, 2009; Yu et al., 2012), and the pH_pzc_ of amorphous ferrihydrite is mostly in the range of 7.6–8.7 (Kosmulski, 2009; Antelo et al., 2010). As a mixture of these compounds, the amorphous FC1 and FC2 show relatively higher pH_pzc_ values. However, the crystalline CuFe_2_O_4_ illustrates a lower pH_pzc_ value, which is consistent with previously reported values (Zhang T et al., 2013; Tu et al., 2014; Sun et al., 2015). For the FC1, a remarkable decrease in pH_pzc_ value has been observed after reaction with As(V) and the pH_pzc_ of As(V)-adsorbed FC1 is about 6.7. Apparently, As (V) is specifically adsorbed by the FC1, since the specific sorption of anions leads to a shift of the pH_pzc_ of adsorbent to a lower pH value (Hsia et al., 1999; Ren et al., 2011). However, a slight decrease in pH_pzc_ of FC1 has been found after reaction with As(III). Commonly, the adsorption of uncharged As(III) species can not result a significant shift in pH_pzc_ of adsorbents (Ren et al., 2011). The slight decrease in pH_pzc_ might be explained as follows. A small part of As (III) adsorbed on the FC1 was oxidized to As(V) by the dissolved oxygen because the experiments were conducted in an open system and the present CuO content might catalyze this reaction. For the FC2, FC3 and FC4, similar phenomena have been observed.

**FIGURE 8:**
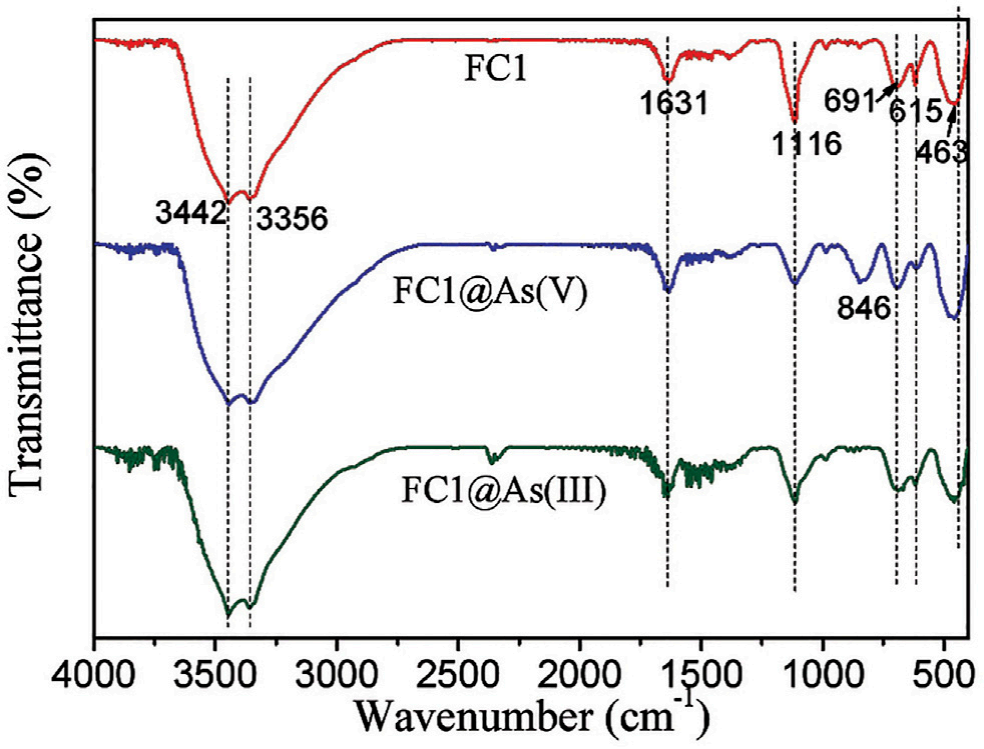
FTIR spectra of Fe-Cu binary oxides before and after arsenic adsorption. Initial arsenic concentration = 10 mg/L, adsorbent dose = 200 mg/L, pH = 7.0 ± 0.1.

FTIR spectra of the FC1 before and after arsenic adsorption are depicted in Figure 8. For the pristine FC1, the peaks at 3,442 and 3,356 cm^−1^ may belong to the vibration of O-H stretching and the peak at 1,631 cm^−1^ may be ascribed to the deformation vibration of water molecules, implying that the surface of FC1 sorbed water molecules through physical adsorption; the peak at 1,116 cm^−1^ may be assigned to the vibration of SO_4_^2-^ (Lefevre, 2004); the three peaks at 691, 615 and 463 cm^−1^ may be ascribed to the overlap of the HO- deformation vibration of ferrihydrite and Cu–O stretching (Kliche and Popovic, 1990). After As (V) adsorption, the intensity of peak at 1,116 cm^−1^ weakens and a new peak appears at 846 cm^−1^, which may be due to the vibration (As-OH) of As-O-M groups (Goldberg and Johnston, 2001). This result suggests that the As (V) is mainly sorbed through the formation of inner-sphere surface complexes. While no significant change has been observed after As (III) adsorption. Similar phenomena were also observed for arsenic adsorption by other metal oxides (Ren et al., 2011).

Based on the analyses of zeta potentials and FTIR sepctra, it could be reasonably concluded that the As (V) is specifically sorbed by the FCBOs via formation of inner-sphere surface complexes, while As (III) is sorbed through formation of both inner- and outer-sphere surface complexes.

## Conclusion

A series of Fe-Cu binary oxides were prepared under different solution pH values. The crystallinity and saturation magnetization of prepared Fe-Cu binary oxide increased with an increase in synthetic pH value. Simultaneously, the morphology of FCBO changed gradually from irregular agglomerate to relatively uniform polyhedron. The adsorption of arsenic on FCBOs is remarkably affected by the surface structure and crystallinity, decreasing as the degree of crystallinity increases. Surface hydroxyl density of FCBOs is an important parameter to evaluate its arsenic adsorption ability. Nevertheless, the adsorption ability may be overestimated if only this parameter is used. As (V) is sorbed by the FCBOs via formation of inner-sphere surface complexes and As (III) is sorbed through formation of both inner- and outer-sphere surface complexes. This investigation provides new insights into structure-performance relationship of FCBOs system, which are beneficial to develop new and efficient sorbents. However, the characterization of FCBOs is still not insufficient in this study and more powerful techniques such as X-Ray Absorption Fine Structure (XAFS) are needed to reveal further the structure-performance relationship of FCBOs system in future study.

## Data Availability

The original contributions presented in the study are included in the article/Supplementary Material further inquiries can be directed to the corresponding authors.
