# Supplementary material for: Efficient Sorption of Arsenic on Nanostructured Fe-Cu Binary Oxides: Influence of Structure and Crystallinity
Source: Front Chem. 2022 Jan 20;9:840446. doi: 10.3389/fchem.2021.840446 (PMC8811158; doi:10.3389/fchem.2021.840446)
Supplement: Supplementary file 1 [file DataSheet1.docx]

**Supplementary Material**

**Efficient sorption of arsenic on nanostructured Fe-Cu binary oxides: influence of structure and crystallinity**

Gaosheng Zhang^1,*^, Zhijing Wu^1^, Qianying Qiu^1^, Yuqi Wang^1, *^

^1^School of Environmental Science and Engineering; Key Laboratory for Water Quality and Conservation of the Pearl River Delta, Ministry of Education, Guangzhou University, Guangzhou 510006, China

*Corresponding author

*E-mail address*: gszhang@gzhu.edu.cn (G. Zhang); [yqwang@gzhu.edu.cn](mailto:yqwang@gzhu.edu.cn) (Y. Wang).

**Batch sorption experiments**

To investigate the influence of pH on arsenic sorption, experiments were carried out by adding 10 mg of Fe-Cu binary oxide into 100-mL glass vessels, containing 50 ml of 10 mg/L arsenic solution. The pH of the solutions was adjusted every four hours with dilute HNO_3_ or/and NaOH solution to designated values during sorption process. The equilibrium pH was measured and the supernatant was filtered through a 0.45 μm membrane after the solutions were mixed for 24 h. Then, the residual arsenic concentration in the supernatants was determined using an inductively coupled plasma mass spectrometry machine (ICP-MS).

For sorption isotherms, the experiments were performed at pH 7.0. The pH of suspensions was adjusted with 0.1 M of NaOH and HNO_3_ during the experiment. Initial arsenic concentration varied from 5 mg/L to 60 mg/L. In each test, 10 mg of Fe-Cu binary oxide was loaded in the 100-mL glass vessel and 50 ml of solution containing differing amounts of arsenic were then added to the vessel. The vessels were shaken on an orbit shaker at 170 rpm for 24 hours at 25 ± 1˚C. Then, all samples were filtered by a 0.45 µm membrane filter and analyzed for arsenic.

**Langmuir and Freundlich models**

$q_{e}=\frac{q_{\max}K_{L}C_{e}}{1+K_{L}C_{e}}$ (1)

$q_{e}=K_{F}C_{e}^{1/n}$ (2)

where q_e_ is the amount of arsenic adsorbed on the solid phase (mg/g), Ce is the equilibrium arsenic concentration in solution phase (mg/L), b is the equilibrium adsorption constant related to the affinity of binding sites (L/mg), q_max_ is the maximum amount of the arsenic per unit weight of adsorbent for complete monolayer coverage, K_F_ is roughly an indicator of the adsorption capacity and 1/n is the heterogeneity factor which has a lower value for more heterogeneous surfaces.
